# Lifestyle intervention to improve sleep quality in Chinese college students: a systematic review

**DOI:** 10.3389/fpsyt.2026.1716523

**Published:** 2026-03-13

**Authors:** Jing Wang, Ismarulyusda Ishak, Fatin Hanani Mazri, Ching Sin Siau, Fengxue Xin, Xiaojuan Wang, Arimi Fitri Mat Ludin

**Affiliations:** 1 Taishan Vocational College of Nursing, Taian, Shandong, China; 2 Center for Toxicology and Health Risk, Faculty of Health Sciences, Universiti Kebangsaan Malaysia, Kuala Lumpur, Malaysia; 3 Center for Healthy Ageing & Wellness, Faculty of Health Sciences, Universiti Kebangsaan Malaysia, Kuala Lumpur, Malaysia; 4 Centre for Community Health Studies, Faculty of Health Sciences, Universiti Kebangsaan Malaysia, Kuala Lumpur, Malaysia; 5 College of Biotechnology and Pharmaceutical Engineering, Nanjing Tech University, Nanjing, Jiangsu, China; 6 Pharmaceutical Bioprosess Technology Division, School of Industrial Technology, Universiti Sains Malaysia, Gelugor, Penang, Malaysia

**Keywords:** China, college students, lifestyle, sleep quality, systematic review

## Abstract

**Objective:**

Sleep quality is a critical determinant of academic performance and overall health among college students. This systematic review aims to synthesize the evidence on the impact of lifestyle interventions on sleep quality among Chinese college students.

**Materials and methods:**

A comprehensive literature search was conducted in four databases including CNKI, PubMed, Web of Science, and Scopus. The review time is from the establishment of the above database to January 2026. Studies were included if they reported on lifestyle interventions and assessed sleep quality using validated measures in Chinese college students. The methodological quality of the included studies was evaluated using the Cochrane Risk of Bias tool.

**Results:**

The search yielded 3993 records, and 16 studies were finally included. The interventions varied in composition but commonly included elements such as physical activity, stress management, and sleep hygiene education. Despite methodological heterogeneity, the collective findings suggest that lifestyle interventions significantly improve sleep quality compared to single or no interventions.

**Conclusion:**

In summary, the lifestyle intervention has a comprehensive and positive impact on the sleep quality of Chinese college students, especially in improving sleep quality, sleep disorders, and daytime dysfunction.

**Systematic review registration:**

https://www.crd.york.ac.uk/PROSPERO/myprospero, identifier CRD42024546680.

## 1 Introduction

In recent years, the problem of poor sleep quality among Chinese college students has aroused wide concern (1). As academic pressure, social media use, and irregular lifestyle habits become more prevalent, an increasing number of college students report sleep-related problems, including insufficient sleep, disrupted sleep patterns, and decreased sleep quality (2–4). This phenomenon is particularly prominent in higher education settings, where sleep problems have been recognized as an important factor influencing academic performance and daily health (5). Notably, healthy lifestyle and behavioral changes have been shown to have a positive impact on improving sleep quality (6). Lifestyle interventions, such as improved dietary habits, regular physical activity, and stress management skills, are effective strategies to improve sleep and promote general health (7–9). These interventions may not only help college students overcome insomnia and other sleep disorders but also improve mental health and cognitive function, ultimately affecting academic performance and life quality.

This study hypothesized that lifestyle interventions, including sufficient physical activity, and effective stress management techniques, can significantly improve sleep quality among college students. In addition, we hypothesized that this multi-component intervention may also enhance the overall health of individuals. This review intended to systematically evaluate and synthesize current research on the association of lifestyle interventions with improved sleep quality and health outcomes in college students. Through this systematic review, we aimed to clarify the effects of various interventions and provide empirical evidence to support the future promotion of effective sleep promotion strategies. The implications of this study extend to promote the overall health of college students by improving sleep quality and providing valuable insights for education and public health policymakers.

## 2 Materials and methods

### 2.1 Literature search strategy

A systematic search was conducted across the following databases: China National Knowledge Infrastructure (CNKI), PubMed, Web of Science, and Scopus. This review focused on intervention in improving sleep quality among Chinese college students. The literature search covered from the initiation up to January 2026. We screened the titles and abstracts of all retrieved records to identify potentially relevant studies. For articles that met the inclusion criteria based on the initial screening, we sought to retrieve the full text. If the full text was not immediately available, we attempted to access it through our institutional library or by contacting the corresponding authors. No language restrictions were imposed. This systematic review was registered in PROSPERO (CRD42024546680) and following the Preferred Reporting Items for Systematic Review and Meta-Analysis (PRISMA) guidelines (10). The general search terms used in this study are summarized in **Table 1**. The complete search strategies, including exact queries, Boolean operators, and specific filters for each database, are detailed in **Supplementary Table 1**.

**Table 1:** Search strings used in this review.

Type	Search	Query
Population	Search string 1	China OR Chinese
	Search string 2	Undergraduate OR Academician OR Students OR College student OR university student OR vocational college student OR undergraduate student OR graduate student OR doctoral student OR master student OR adolescent
Intervention	Search string 3	Intervention OR intervention treatment OR comprehensive intervention measures OR intervention effect OR intervention method OR treatment OR treatment method OR treatment plan OR comprehensive treatment
Comparison	Search string 4	No intervention OR single intervention
Outcome	Search string 5	Sleep quality OR sleep hygiene OR sleep habit OR sleep well

### 2.2 Inclusion criteria

The study’s inclusion criteria were as follows: (1) Participants were Chinese college students aged 18 years or older, (2) the research designs included intervention studies, quasi-experimental designs, and randomized controlled trials, (3) the experimental group received one or more of the interventions to improve sleep quality, and (4) the primary outcome was the sleep quality score.

### 2.3 Exclusion criteria

The exclusion criteria were as follows: (1) Research indicators that do not align with the inclusion criteria, and (2) reviews, case studies, survey analyses, conference abstracts, and unrelated literature.

### 2.4 Literature screening and data extraction

Two evaluators (JW and XW) independently reviewed the titles, abstracts, and full texts of the literature retrieved from each database to determine eligibility based on the aforementioned criteria. In cases of disagreement, the original articles were re-examined, and consensus was reached through discussion. Data were extracted from the above-selected literature, including details such as author, year of publication, sample size, age, intervention measures, intervention duration, and outcome indicators.

### 2.5 Study selection

**Figure 1:**
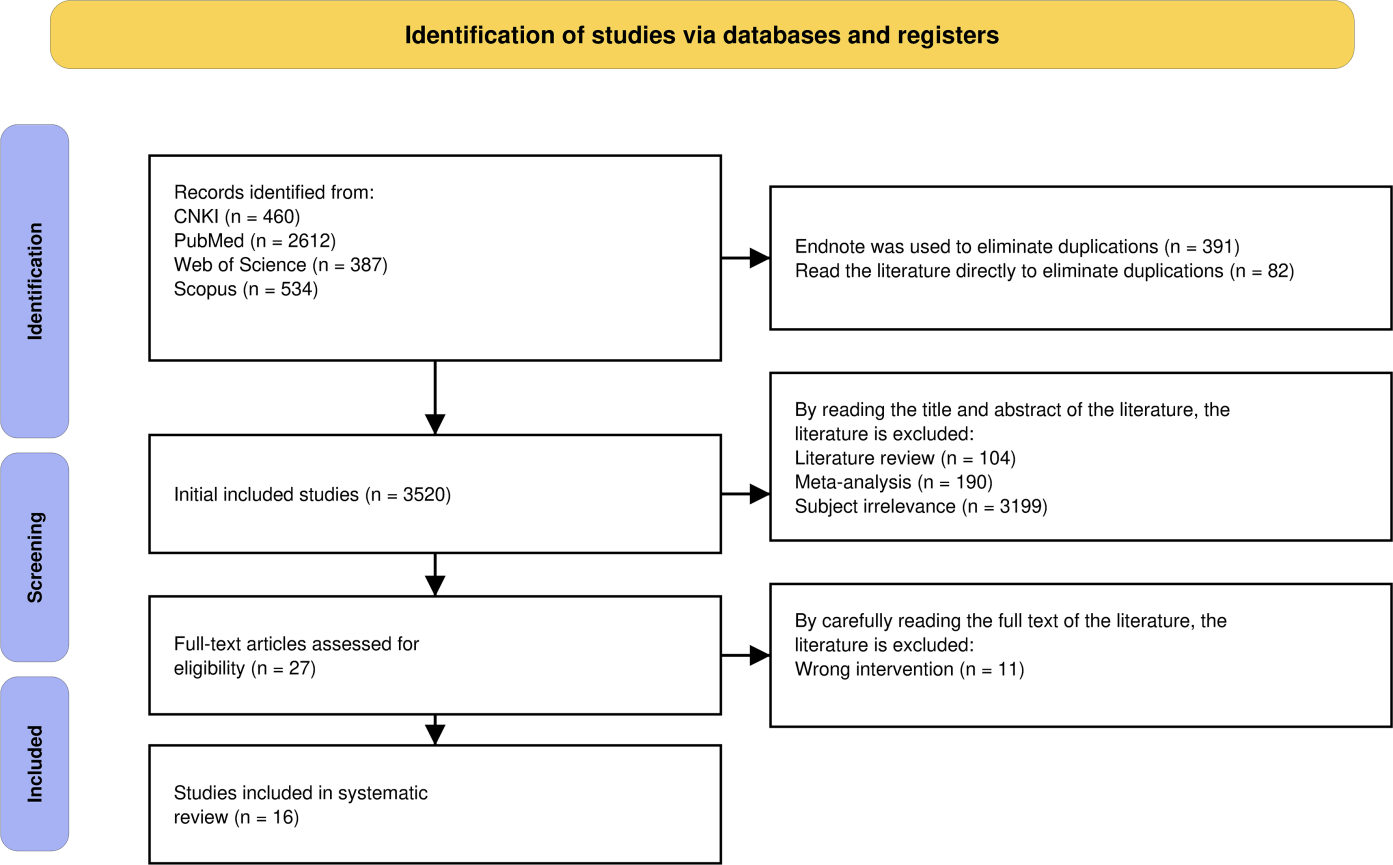
PRISMA flow chart.

A total of 3993 articles were retrieved from the selected databases: 460 from CNKI, 2612 from PubMed, 387 from Web of Science, and 534 from Scopus. After identifying 473 duplicates using Endnote 20, the titles and abstracts of the remaining articles were reviewed, excluding 104 literature reviews, 190 meta-analyses, and 3199 articles unrelated to the subject. Upon further examining the full text, 11 articles were excluded for not meeting the outcome indicators. Finally, 16 articles were included in this systematic review (11–26) (**Figure 1**).

### 2.6 Literature quality evaluation

The quality of selected literature was evaluated by two authors using the Cochrane Risk of Bias tool, and the evaluating factors included randomization method, allocation concealment, blinding, result data, selective reporting, and other biases. Each study was rated as High risk, Low risk, and Unclear risk. Disagreements were resolved through discussion with a third author (FX).

### 2.7 Data synthesis

Due to the significant heterogeneity across the included studies regarding intervention types (ranging from aerobic exercise to mindfulness and acupressure), intervention durations (2 to 16 weeks), and control conditions, a quantitative meta-analysis was deemed inappropriate. Consequently, the study adopted a narrative synthesis approach following the Synthesis Without Meta-analysis (SWiM) reporting guidelines. Studies were grouped by intervention category (Physical/Athletic Training vs. Mind-Body Interventions) to facilitate a structured comparison of findings. We prioritized the reporting of intervention characteristics and outcome consistency to assess the validity of the evidence.

## 3 Results

### 3.1 Study characteristics

The 16 included studies were published between 2010 and 2024, covering multiple provinces in China, including Xinjiang, Guangdong, Sichuan, Hubei, Beijing, Shanghai, Guizhou, Northeast China, Hunan, Zhejiang, Hebei, and Shanxi. Except for the studies by Chen X et al. (21) and Zhang et al. (22) all other studies used randomized controlled trials (RCTs). The number of participants in each study ranged from 19 to 364. The intervention period varied between 2 and 12 weeks, with most studies having an intervention period of 4 or 8 weeks. All studies used the Pittsburgh Sleep Quality Index (PSQI) as the tool to assess sleep quality. The detailed information is shown in **Table 2**.

**Table 2:** Study characteristics.

Author, year	Area, country	Study design	Number of participants		Duration of intervention	Sleep measures
			Intervention group	Control group		
Bi et al., 2024 (14)	Xinjiang, China	RCT	15/15/15	16	10 weeks	PSQI
Chen X et al., 2017 (21)	China	Quasi-experimental designs	32	40	8 weeks	PSQI
Chen Y et al., 2022 (20)	Guangdong, China	RCT	9	10	8weeks	PSQI
Ding et al., 2024 (18)	Sichuan, China	RCT	27	30	2 weeks	PSQI
Gao et al., 2022 (16)	Hubei, China	RCT	44	45	12 weeks	PSQI
Guo et al., 2023 (19)	Sichuan, China	RCT	20	20	4weeks	PSQI
Ji et al., 2022 (11)	Northeastern, China	RCT	66/64	67	6 weeks	PSQI
Li M et al., 2022 (17)	Beijing, China	RCT	193	171	12 weeks	PSQI
Li X et al., 2022 (15)	Shanghai and Guizhou, China	RCT	39/37	21	3 weeks	PSQI
Qiu et al., 2023 (12)	China	RCT	19/18/19	19	16 weeks	PSQI
Wang et al., 2019 (26)	Hunan, China	RCT	37	36	4 weeks	PSQI
Xu et al., 2022 (13)	Zhejiang, China	RCT	43	43	12 weeks	PSQI
Yin et al., 2011 (23)	Hebei, China	RCT	30/30/30	30	4 weeks	PSQI
Zhang et al., 2010 (22)	Guangdong, China	Quasi-experimental designs	60	/	12 weeks	PSQI
Zheng et al., 2019 (24)	China	RCT	50	49	8 weeks	PSQI
Zhou et al., 2014 (25)	Shanxi, China	RCT	30	30	12 weeks	PSQI

RCT, randomized controlled trial.

### 3.2 Quality assessment result

**Figure 2:**
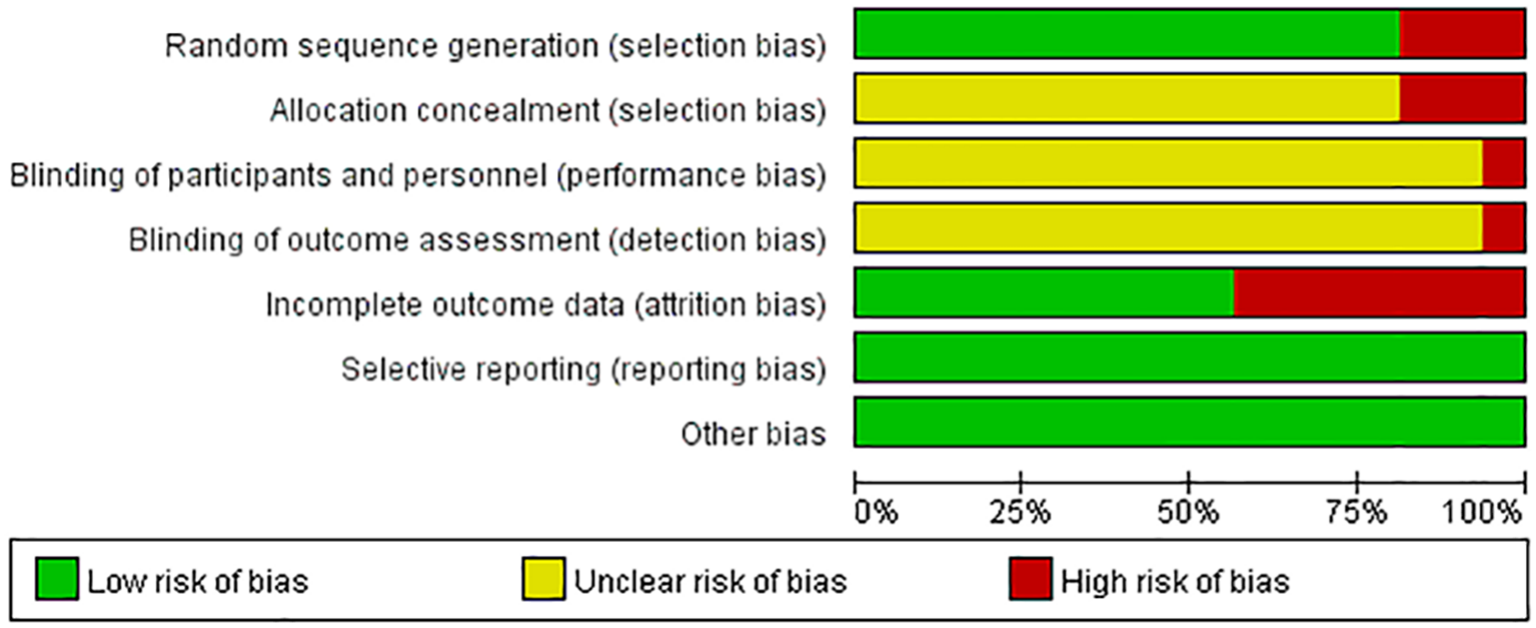
Bias risk bar chart.

**Figure 3:**
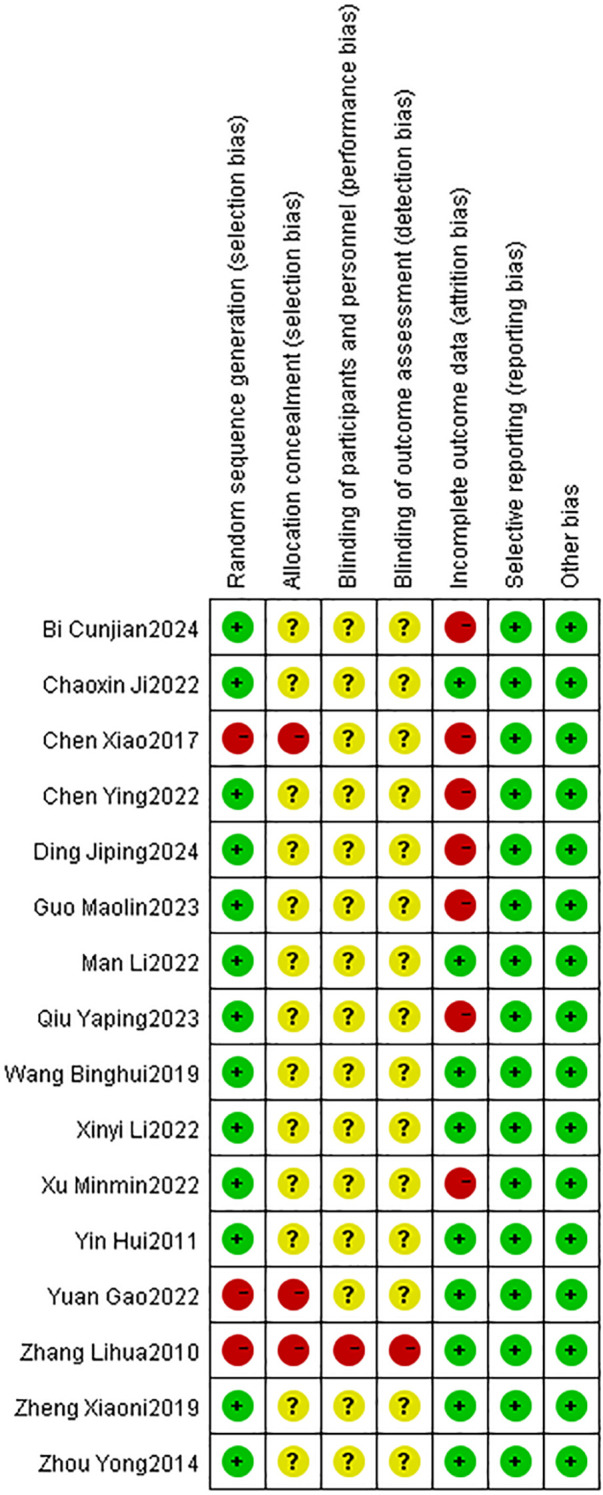
Bias risk graph.

Cochrane risk bias plots were plotted to evaluate the quality of the included literature. Among the 16 studies included, 10 studies only mentioned randomness, 1 used random number table method, 1 study employed random drawing method, and 1 used SPSS software for randomization. These 13 studies were evaluated as low risk in terms of “random method”. However, 3 studies did not use random grouping and was evaluated as high risk in terms of “random method”. Regarding “allocation hiding”, 13 studies did not mention it, and 3 studies did not use allocation hiding, resulting in a high risk for that study. only one of the 16 studies provided information on “blinded assessment of implementers, participants, and outcome assessors”, and was therefore rated as low risk, while the other studies had unknown risk on this criterion. Seven studies experienced participant dropout and were rated as high risk in terms of “incomplete outcome data”. The remaining 9 studies had complete outcome data, so they were rated as low risk in this category. All studies were rated as low risk for “selective reporting” and “other bias” (**Figures 2**, **3**).

### 3.3 Included study characteristics

The 16 studies included in this review were categorized into two major types of interventions: physical and athletic training and mind-body interventions. Nine studies reported on the impact of physical and athletic training on college students’ sleep quality, and seven studies examined mind-body interventions (**Table 3**).

**Table 3:** Characteristics of intervention and outcome.

Type	Author	Intervention	Outcome
Physical and athletic training	Bi et al., 2024 (14)	The intervention groups include AE&RE, AE&BE, and RE&BE. Classroom exercises for 50 min, every Monday, Wednesday, and Friday, and music therapy is cross-over with classroom exercises.	All intervention groups significantly reduced PSQI scores compared to baseline: AE&RE (9.21 ± 3.79 to 6.11 ± 2.99, *P<0.05*), AE&BE (8.14 ± 3.21 to 5.36 ± 1.97, *P<0.01*), RE&BE (8.99 ± 2.71 to 7.01 ± 2.32, *P<0.05*). Control group showed no significant change (11.01 ± 3.32 to 10.12 ± 3.01).
	Gao et al., 2022 (16)	Aromatherapy yoga or yoga for 90 min every week.	Total PSQI scores did not change significantly in either Aromatherapy Yoga (6.86 ± 2.54 to 6.66 ± 2.69) or Yoga-only groups (6.56 ± 2.40 to 6.98 ± 2.80, *P>0.05*). However, Aromatherapy Yoga significantly improved the “sleep disturbance” subscore (1.30 ± 0.46 to 1.07 ± 0.50, *P=0.04*).
	Ji et al., 2022 (11)	Team sports: 10 min warm-up + 40 min basketball game + 10 min relaxation. Individual sports: approx. 45 min (2 km run + strength training + 5 min relaxation).	Both interventions improved sleep quality more than control. Team sports (OR = 7.98, 95% CI: 6.69–19.98, *P=0.003*) and individual sports (OR = 7.32, 95% CI: 5.35–18.22, *P=0.005*) showed higher odds of improvement compared to control. No significant difference between the two sports modes.
	Qiu et al., 2023 (12)	Three times per week, each session lasting 60 min (aerobic training and/or resistance training).	Post-intervention PSQI scores were significantly lower in all exercise groups compared to control (9.03 ± 3.67): AT (6.82 ± 2.98), RT (7.40 ± 3.11), and AT+RT (5.24 ± 1.95). The Combined group showed the lowest scores (F = 4.536, *P=0.037*).
	Xu et al., 2022 (13)	Circulatory resistance training: initial phase (1st month) + advanced phase (2nd–3rd month).	Post-intervention PSQI scores in the CRT group (6.41 ± 1.38) were significantly lower than the control group (7.81 ± 2.57) (t=3.443, *P<0.001*). The CRT group showed significant reductions in all PSQI sub-dimensions (*P<0.05*).
	Yin et al., 2011 (23)	Mass Exercise I, II, and Yoga Stretch Stretching is an AE, which is carried out on Mondays, Wednesdays, and Fridays every week and lasts for 45–60 min each time; the auricular points therapy group takes subcortical, heart, kidney, Shenmen, sympathetic, endocrine and other points on the ear were pressed and kneaded 3~4 times a day, 2~5 min/time, and the pressure was strengthened for 1~2 min before going to bed, alternating between the two ears, and the points were replaced once every 3~4 d, and the period of 7 d was one course of treatment.	Post-intervention PSQI scores were significantly lower in all intervention groups compared to control (9.17 ± 1.27, *P<0.05*): Aerobic (6.83 ± 1.27), Auricular (6.52 ± 1.28, and Combined (6.00 ± 1.25). The Combined group showed the best improvement.
	Zhang et al., 2010 (22)	Yoga physical therapy with 10 poses, practiced 5 times a week for 60 min each time.	Significant within-group improvement: PSQI total scores dropped from 8.37 ± 4.00 at baseline to 2.84 ± 1.80 post-intervention *(P<0.01*). The recovery rate was 60%.
	Zheng et al., 2019 (24)	Playing team sports such as basketball and badminton in small groups for more than 60 min a day.	Post-intervention PSQI scores were significantly lower in the intervention group (5.92 ± 2.77) compared to the control group (8.18 ± 2.63) (t=3.885, *P<0.001*).
	Zhou et al., 2014 (25)	The experimental group underwent Five-Animal Exercises, 2 times a day, 6 days a week, with 2–3 repetitions of each movement; the control group underwent psychotherapy.	Both groups improved, but the Five-Animal Exercises group (9.7 ± 1.2) had significantly lower post-intervention PSQI scores than the Psychotherapy group (10.7 ± 2.6) (*P<0.01*).
Mind-body interventions	Chen X et al., 2017 (21)	Mindfulness meditation training is practiced once a week for 45–60 min.	The mindfulness group had significantly lower post-test PSQI scores (5.41 ± 1.50) compared to the control group (6.60 ± 2.40) (F = 6.67, *P<0.05*).
	Chen Y et al., 2022 (20)	Members of the intervention group received ACT once a week on Thursdays for 120 min; members of the control group received mental health education for college students once a week for 120 min.	Post-intervention PSQI scores in the ACT group (6.67 ± 3.61) were significantly lower than the control group (10.20\ ± 2.97) (t=−2.340, *P=0.032*).
	Ding et al., 2024 (18)	The treatment group received ear acupoint pressing combined with five-tone therapy for 7 days as one course of treatment, for a total of 2 courses.	The intervention group showed a greater reduction in PSQI scores (reduction of 5.48 ± 2.21) compared to the control group (reduction of 2.17 ± 2.67) (t=5.08, *P<0.001*).
	Guo et al., 2023 (19)	Mindfulness yoga twice a week.	Significant interaction effect on PSQI scores (F = 32.730, *P<0.01*). The intervention group improved from 9.90 ± 2.67 to 5.85 ± 2.11, while the control group showed no significant improvement (8.85 ± 0.81 to 8.15 ± 1.53).
	Li M et al., 2022 (17)	Approx. 30 min (health education, establishing dormitory sleep rules, use of earplugs and eye masks).	Linear mixed-effects models showed a significant intervention effect on PSQI total score (β=−0.67, P = 0.012). The intervention group significantly reduced PSQI scores (5.61 ± 2.35 to 5.12 ± 2.43), while the control group did not change.
	Li X et al., 2022 (15)	CBT intervention or conventional sleep education 10–15 min every day.	Both dCBT-i (8.08 ± 0.44 to 5.43 ± 0.35) and conventional education (8.46 ± 0.50 to 5.29 ± 0.40) significantly improved sleep quality (*P<0.001*). Healthy controls showed deteriorated sleep (2.50 ± 0.62 to 5.39 ± 0.49) during the same period.
	Wang et al., 2019 (26)	Snap needles were buried into the acupoints and left in place for 48 h. Pressure was applied for 2 min in the morning, midday, and evening every day, and the needles were buried three times a week, with a rest on Sundays; during the period of burying the needles the subjects set up a 30-min timed music at bedtime.	The combination group (Snap-needle + Music) achieved significantly lower post-intervention PSQI scores (3.43 ± 1.41) compared to the single therapy group (7.59 ± 1.76) (t=10.021, *P<0.01*).

AE&RE, aerobic exercise and resistance exercise; AE&BE, aerobic exercise and body exercise; RE&BE, resistance exercise and body exercise; ACT, Acceptance and Commitment Therapy; CBT, Cognitive Behavioral Therapy.

#### 3.3.1 Physical and athletic training

Nine studies evaluated the impact of physical interventions. Bi et al. (14) reported that different exercise combinations significantly reduced PSQI scores compared to baseline, with the “Aerobic & Body Exercise” group showing the most substantial decrease from 8.14 ± 3.21 to 5.36 ± 1.97 *(P<0.01)*. Similarly, Qiu et al. (12) found that while aerobic and resistance training alone improved sleep, the combined training group achieved the lowest post-intervention PSQI score of 5.24 ± 1.95, significantly lower than the control group’s 9.03 ± 3.67 *(P = 0.037)*. In terms of traditional exercises, Zhou et al. (25) demonstrated that Five-Animal Exercises were more effective than psychotherapy, with post-intervention scores of 9.7 ± 1.2 vs. 10.7 ± 2.6 *(P<0.01)*. Xu et al. (13) found that Circuit Resistance Training (CRT) significantly lowered PSQI scores compared to controls (6.41 ± 1.38 vs. 7.81 ± 2.57, *P<0.001*). Ji et al. (17) highlighted that both team sports (OR = 7.98) and individual sports (OR = 7.32) significantly improved the odds of better sleep quality compared to controls. Zhang et al. (22) observed a significant within-group improvement following yoga therapy, with mean PSQI scores dropping from 8.37 ± 4.00 to 2.84 ± 1.80 (*P<0.01*). However, Gao et al. (16) noted that while Aromatherapy Yoga improved specific sleep disturbance subscores (*P=0.04*), the total PSQI score did not show a statistically significant difference compared to the yoga-only group (*P>0.05*).

#### 3.3.2 Mind-body interventions

Seven studies examined mind-body interventions. Chen X et al. (21) reported that mindfulness meditation led to significantly lower post-test PSQI scores compared to the control group (5.41 ± 1.50 vs. 6.60 ± 2.40, F = 6.67, *P<0.05*). Similarly, Guo et al. (19) found a significant interaction effect for mindfulness yoga (F = 32.730, *P<0.01*), with the intervention group improving from 9.90 ± 2.67 to 5.85 ± 2.11. Chen Y et al. (20) demonstrated that Acceptance and Commitment Therapy (ACT) significantly reduced PSQI scores compared to controls (6.67 ± 3.61 vs. 10.20 ± 2.97, *P=0.032*). Ding et al. (18) found that combining ear acupoint pressing with five-tone therapy resulted in a significantly greater reduction in PSQI scores (−5.48 ± 2.21) compared to the control group (−2.17 ± 2.67) (*P<0.001*). Regarding environmental interventions, Li M et al. (17) showed that a dormitory-based intervention significantly decreased PSQI scores (β = −0.67, *P=0.012*). Additionally, Wang et al. (26) found that combining snap-needle therapy with music therapy was more effective (PSQI: 3.43 ± 1.41) than snap-needle therapy alone (7.59 ± 1.76) *(P<0.01*).

## 4 Discussion

The current systematic review consolidates evidence from 16 studies that explore the effects of various interventions on sleep quality among college students in China. These interventions, categorized into physical/athletic training and mind-body practices, demonstrate significant efficacy in improving sleep, albeit through distinct mechanisms. The findings align with and expand on previous research, underscoring the diversity and potential effectiveness of intervention strategies. A noteworthy aspect of the included studies is their broad geographic representation, covering multiple provinces across China. This diversity ensures a more generalized understanding of intervention effectiveness across cultural and environmental contexts. Prior studies conducted in specific regions or populations may have overlooked such variability, and the present review addresses this gap by incorporating a wider participant pool. Furthermore, included studies spanning 14 years (2010–2024) provides a robust longitudinal perspective on evolving intervention methodologies and their relative successes in the Chinese context.

Physical exercise interventions, particularly those focused on aerobic and resistance exercises, have long been recognized for their positive impact on sleep quality. Physical activities like aerobic and resistance exercises can improve sleep quality by enhancing cardiovascular function and alleviating symptoms of anxiety and depression (27, 28). Mechanistically, exercise may improve sleep quality by increasing energy expenditure, promoting endorphin secretion, or regulating body temperature. Furthermore, it may lower inflammatory cytokine levels, contributing to improved sleep (29). Consistent with the findings of this review, Huang et al. (30) noted that engaging in physical exercise significantly improved sleep parameters, including sleep efficiency and sleep latency. This review supports such findings, with several studies (e.g., 12–14) reporting improvements in sleep quality through aerobic and resistance training. Additionally, the review emphasizes that combined physical activities (such as aerobic and resistance training) tend to be more effective than single-mode exercises, aligning with recent research exploring multi-component exercise programs to enhance sleep outcomes (31). Aside from Western exercise modalities, Zhou et al. (25) demonstrate that traditional Chinese exercises like Five-Animal Exercises also significantly improve sleep quality. This suggests that culturally tailored physical interventions hold great potential, especially for populations with strong traditional practices. This provides a pathway for future research to integrate cultural and exercise science.

On the other hand, mind-body interventions, including mindfulness therapy, CBT, and ACT, have also been shown to significantly improve sleep quality among college students. These interventions work by enhancing emotional regulation and promoting relaxation, both of which are crucial for initiating and maintaining sleep (32–34). Research by Chen Y et al. (20) indicated that ACT significantly reduced PSQI scores, underscoring its efficacy in addressing cognitive and emotional barriers to sleep. Similarly, Li X et al. (15) observed that CBT was more effective than traditional sleep education, demonstrating that cognitive restructuring and behavioral adjustments are more effective in addressing underlying sleep problems, especially in students with anxiety and stress-related sleep issues. Furthermore, this review emphasizes the benefits of combining complementary therapies such as auricular acupressure and Five-Tone Therapy (18) to improve sleep quality. Previous studies have noted that acupressure can reduce inflammation, lower blood pressure, and enhance neural system responses (35, 36). Lin et al. found that a combination of acupoint treatments was more effective than single acupoint therapy in improving sleep quality (37). These traditional Chinese medicine-based therapies provide new avenues for improving sleep quality.

Interventions targeting both physical and psychological health are essential, and the findings of this review highlight the importance of interventions aimed at improving sleep quality, particularly among Chinese college students. Physical exercise and mind-body interventions offer a comprehensive toolkit for enhancing students’ sleep quality, which is especially important in the unique cultural and academic stress context of China. The evidence presented in this review supports the implementation of multifaceted interventions that address both physiological and psychological aspects of sleep quality, offering promising directions for future research and clinical applications. Further studies should aim to elucidate the specific mechanisms by which these interventions interact, particularly about stress reduction, immune modulation, and circadian rhythm regulation.

An evident gap identified from this review is the fact that none of the included study employed nutritional intervention. Previous studies showed that Certain foods can enhance or disrupt sleep. For instance, heavy meals, particularly those high in carbohydrates, can lead to increased sleepiness, while lighter meals may facilitate better sleep onset (38). Nutritional deficiencies, such as low magnesium or vitamin D, have been associated with poor sleep quality (39). The timing of meals also plays a role; late-night eating can interfere with circadian rhythms, associated with poorer sleep quality (40) The lack of evidence in this matter warrant further investigation. Given the close link between nutrition and sleep, future studies should incorporate nutritional assessments and interventions to provide a more holistic lifestyle approach for improving sleep quality in college students.

This study has certain limitations that require further improvement and refinement. (1) The follow-up durations varied across the included studies, which may have influenced the data measurement outcomes. (2) The sample size in some studies was relatively small. Future research should focus on increasing sample sizes to reduce bias, extending follow-up periods, and utilizing objective sleep monitoring tools to assess sleep quality more accurately.

## 5 Conclusions

In conclusion, this review provides valuable insights into the efficacy of physical, athletic, and mind-body interventions for improving sleep quality among college students in China. Despite some methodological limitations, the findings highlight the potential of these interventions to mitigate sleep disturbances and improve overall well-being in this population. Future research should focus on refining these interventions and exploring their long-term impact to better inform public health strategies targeting sleep health in college students.

## Data Availability

The original contributions presented in the study are included in the article/**Supplementary Material**. Further inquiries can be directed to the corresponding author.
