# Supplementary material for: Lifestyle intervention to improve sleep quality in Chinese college students: a systematic review
Source: Front Psychiatry. 2026 Mar 13;17:1716523. doi: 10.3389/fpsyt.2026.1716523 (PMC13021658; doi:10.3389/fpsyt.2026.1716523)
Supplement: Supplementary file 1 [file Table1.docx]

**Search strategies in CNKI（1288）**

| Search |  | Query |
| --- | --- | --- |
| #1  #2  #3  #4 |  | 学生 + 大学生 + 高职院校学生 + 大专生 + 本科生 + 研究生 + 博士生 + 硕士生 + 中专生 + 青少年  睡眠 + 睡觉 + 睡眠质量 + 睡眠品质 + 睡觉质量 + 睡觉品质  干预 + 干预治疗 +综合丅预 + 干预措施 +干预效果 +干预方法 + 治疗 + 治疗方法 +治疗方案 + 综合治疗  #1 AND #2 AND #3 |

**Search strategies in pubmed（997）**

| Search |  | Query |
| --- | --- | --- |
| #1  #2  #3  #4 |  | (China) OR (Chinese)  ((((((((((Undergraduate) OR (Academician)) OR (Students)) OR (College student)) OR (university student)) OR (vocational college student)) OR (undergraduate student)) OR (graduate student)) OR (doctoral student)) OR (master student)) OR (adolescent)  (((((((((((((((Qualities, Sleep) OR (Quality, Sleep)) OR (Sleep Qualities)) OR (Sleep Hygiene)) OR (Sleeping Habits)) OR (Sleep Habits)) OR (Habit, Sleep)) OR (Habits, Sleep)) OR (Sleep Habit)) OR (Sleeping Habit)) OR (Habit, Sleeping)) OR (Habits, Sleeping)) OR (sleep quality)) OR (Sleeping quality)) OR (quality of sleep)) OR (Sleep well)  ((((((((Intervention) OR (intervention treatment)) OR (comprehensive intervention measures)) OR (intervention effect)) OR (intervention method)) OR (treatment)) OR (treatment method)) OR (treatment plan)) OR (comprehensive treatment) |

**Search strategies in web of science（373）**

| Search |  | Query |
| --- | --- | --- |
| #1  #2  #3  #4 |  | (China) OR (Chinese)  ((((((((((Undergraduate) OR (Academician)) OR (Students)) OR (College student)) OR (university student)) OR (vocational college student)) OR (undergraduate student)) OR (graduate student)) OR (doctoral student)) OR (master student)) OR (adolescent)  (((((((((((((((Qualities, Sleep) OR (Quality, Sleep)) OR (Sleep Qualities)) OR (Sleep Hygiene)) OR (Sleeping Habits)) OR (Sleep Habits)) OR (Habit, Sleep)) OR (Habits, Sleep)) OR (Sleep Habit)) OR (Sleeping Habit)) OR (Habit, Sleeping)) OR (Habits, Sleeping)) OR (sleep quality)) OR (Sleeping quality)) OR (quality of sleep)) OR (Sleep well)  ((((((((Intervention) OR (intervention treatment)) OR (comprehensive intervention measures)) OR (intervention effect)) OR (intervention method)) OR (treatment)) OR (treatment method)) OR (treatment plan)) OR (comprehensive treatment) |

**Search strategies in scopus（493）**

| Search |  | Query |
| --- | --- | --- |
| #1  #2  #3  #4 |  | (China) OR (Chinese)  ((((((((((Undergraduate) OR (Academician)) OR (Students)) OR (College student)) OR (university student)) OR (vocational college student)) OR (undergraduate student)) OR (graduate student)) OR (doctoral student)) OR (master student)) OR (adolescent)  (((((((((((((((Qualities, Sleep) OR (Quality, Sleep)) OR (Sleep Qualities)) OR (Sleep Hygiene)) OR (Sleeping Habits)) OR (Sleep Habits)) OR (Habit, Sleep)) OR (Habits, Sleep)) OR (Sleep Habit)) OR (Sleeping Habit)) OR (Habit, Sleeping)) OR (Habits, Sleeping)) OR (sleep quality)) OR (Sleeping quality)) OR (quality of sleep)) OR (Sleep well)  ((((((((Intervention) OR (intervention treatment)) OR (comprehensive intervention measures)) OR (intervention effect)) OR (intervention method)) OR (treatment)) OR (treatment method)) OR (treatment plan)) OR (comprehensive treatment) |
